# Gender differences in the sleep variables contributing to excessive daytime sleepiness among patients with obstructive sleep apnea

**DOI:** 10.1007/s11325-020-02276-x

**Published:** 2021-01-19

**Authors:** Eliya Honig, Amit Green, Yaron Dagan

**Affiliations:** 1 Faculty of Medicine, Hebrew University of Jerusalem, Jerusalem, Israel; 2 The Sleep and Fatigue Institute, Assuta Medical Center, 96 Yigal Alon Street, 67891 Tel Aviv, Israel; 3 The Research Institute of Applied Chronobiology, The Academic College of Tel-Hai, 1220800 Tel Hai, Israel

**Keywords:** Sex factors, Polysomnography, Sleep apnea, Obstructive

## Abstract

**Purpose:**

Excessive daytime sleepiness (EDS) is a main symptom in patients with obstructive sleep apnea (OSA); however, patients with OSA have significant variability in their reported EDS which cannot be fully explained by the apnea-hypopnea index (AHI). The purpose of this study was to investigate gender differences regarding the sleep test variables contributing to excessive daytime sleepiness.

**Methods:**

Retrospective study of 578 men and 270 women with suspected OSA who underwent home overnight sleep test. We assessed the correlation between sleep test variables and EDS, using the Epworth Sleepiness Scale (ESS).

**Results:**

Among the group of men, correlation was found between ESS to BMI (*r* = .107, *p* = .010), AHI (*r* = .158, *p *< .001), number of apneas (*r* = .129, *p* = .002), number of hypopneas (*r* = .115, *p* = .006), number of blood oxygen desaturations (*r* = .145, *p * < .001), and percent of time the blood oxygen saturation was under 90% (*r* = .130, *p* = .002). However, among the group of women, no significant correlation was found between any of the sleep test parameters or BMI to ESS. Among the group of women, a negative correlation was found between age and EDS (*r* = − .208, *p* < .001).

**Conclusion:**

Men showed correlations between sleep test variables and EDS, while women did not show such correlations. The results suggest that men’s sleepiness is more influenced by OSA and sleep variables compared to women. To our knowledge, this is the first study which shows difference between genders in the influence of sleep variables and OSA on EDS.

Obstructive sleep apnea syndrome (OSAS) is characterized by repeated obstructions of upper airway during sleep. This medical condition is often accompanied by blood oxygen desaturations and by sleep fragmentation.

It is acceptable to grade OSAS severity by the measurement of the number of apnea events (complete airway obstruction of at least 10 s) and hypopnea events (airflow decrease of 50%) per hour during sleep, which is called apnea-hypopnea index (AHI). A disadvantage of AHI in predicting OSA severity is that it does not reflect important factors such as the duration of apneas and hypopneas, and number and duration of blood desaturations.

Excessive daytime sleepiness (EDS) is frequently present in patients with OSAS and is considered a major symptom of the syndrome. If present, it is implicated in decreased quality of life, and may cause poor outcomes. Patients with increased daytime sleepiness have been more likely to report poorer mental health, poorer cognitive function, reduced productivity, and higher rates of work and car accidents [1–4]. However, previous studies have shown conflicting results regarding the correlation between the severity of OSAS according to AHI and the extent of EDS. EDS is not universally present in patients with OSA and sleepiness varies significantly between patients with the same OSA severity according to AHI [5–9]. There is limited data regarding the pathogenesis of sleepiness among patients with OSA, and mechanisms explaining the difference between patients remain poorly understood.

The male-to-female ratio in patients with OSA is estimated between 3:1 and 5:1 in the general population [5, 10, 11]. It has been reported that the clinical manifestations of OSA differ in men and women [12–16]. Mechanisms explaining the phenotype differences between genders are not fully understood. Previous studies that researched the contributing factors to EDS by examining correlations between sleep study parameters and EDS did not evaluate genders separately [17].

The purpose of this study was to characterize gender differences in the contributors of daytime sleepiness. Since excessive daytime sleepiness can be only partially explained by AHI, we hypothesized that more sleep parameters are required to evaluate the predictors of EDS, in addition to AHI. We evaluated the correlation between sleep test variables to EDS, separately for men and women. In addition, mean Epworth Sleepiness Scale (ESS) scores of men and women were compared.

## Methods

### Patient selection

We examined retrospectively the data of 858 patients (578 men, 280 women), who were referred to the Sleep Medicine Institute at Assuta Medical Centers in Israel. All patients completed the ESS questionnaire and underwent home sleep testing. Patients included in the study were over 18 years old, did not have a previous diagnosis of OSAS, and underwent a sleep study for the first time.

### Sample size assessment

The sample size assessment was made based on the expected strength of the relationship between the ESS questionnaire and the time that blood oxygen saturation was less than 90%.

Assumptions included a correlation of at least 0.5, a significance of 5% (unidirectional), and intensity of 80%.

At least 111 subjects were required to demonstrate that this correlation was significantly greater than any sample of significance of 0.3 or less. In this study, we examined 858 patients.

### EDS evaluation, Epworth Sleepiness Scale

Subjective sleepiness was measured using the ESS questionnaire. The questionnaire is considered a reliable method to estimate EDS, and was found to correlate significantly with MSLT, the objective method for the measurement of EDS [18]. We used ESS because it is a reliable and simple method that enables use on a big sample and has been used in previous studies, enabling comparison of the results.

The ESS is composed of 8 questions about the patient’s tendency to fall asleep in common daily situations. Patients were asked to answer each question on a scale of 0 to 3. The total score of a completed ESS questionnaire ranges between 0 and 24. A score of 10 or above suggests excessive daytime sleepiness [18, 19].

### Sleep test evaluation

All participants underwent a home sleep study with SOMNO-TOUCH device. During the sleep study, data of oronasal airflow by nasal canula, snoring, sleep position, chest and abdomen respiratory movement, and pulse oximetry measurements were recorded.

Apnea was defined as absence of airflow for > 10 s. Hypopnea was defined as any airflow reduction of > 50% that lasted for > 10 s. AHI was defined as the sum of apneas and hypopneas events per hour of sleep. Oxygen desaturation was defined as a decrease of 4% in blood oxygen for > 4 s. Sleep data processing was performed by skilled and trained sleep technicians in accordance with the AASM criteria (AASM, 2007).

### Statistical analysis

We calculated the Pearson correlation between the variables in the study in order to identify significant variables. The variables that were checked for correlation with ESS were BMI, sleep duration, AHI, number of apneas, number of hypopneas, mean duration of apnea, mean duration of hypopnea, number of blood oxygen desaturations, percentage of time that the blood oxygen was under 90%, percentage of time of snoring.

All statistical analyses were performed using SPSS, version 25 (SPSS Inc., Chicago, IL, USA).

## Results

Tables 1, 2, and 3 present the correlations between polysomnographic variables and ESS. In the whole sample (men and women), correlation was found between ESS and BMI (*r* = .104, *p* = .002), number of apneas (*r* = .118, *p* = .001), number of hypopneas (*r* = .099, *p* = .004), AHI (r = .142, *p* = .000), number of desaturations (*r* = .124, *p* = .000), and percentage of the time blood oxygen saturation was < 90% (*r* = .103, *p* = .002). A negative correlation was found between age and ESS (*r* = − .109, *p* = .001).

**Table 1:** Whole sample. Correlations between ESS and sleep study variables

	Age	BMI	No. ofapneas	No. of hypopneas	AHI	Max. dur.of apnea	Max. dur.of hypopnea	Mean dur.of apnea	Mean duration of hypopnea	No. ofdesaturations	% time Sat < 90%	% time Snoring
ESS	− *.109*	*.104*	*.118*	*.099*	*.142*	.070	.018	.057	.020	*.124*	*.103*	.068

The significance is 5%

**Table 2:** Men’s group. Correlations between ESS and sleep study variables

	Age	BMI	No. ofapneas	No. ofhypopneas	AHI	Max. dur.of apnea	Max. dur.of hypopnea	Mean dur.of apnea	Mean durationof hypopnea	No. of desaturations	% time Sat < 90%	% time Snoring
ESS	− .057	*.107*	*.129*	*.115*	*.158*	.073	.042	.074	.037	*.145*	*.130*	.079

The significance is 5%

**Table 3:** Women’s group. Correlations between ESS and sleep study variables

	Age	BMI	No. ofapneas	No. ofhypopneas	AHI	Max. dur.of apnea	Max. dur. of hypopnea	Mean dur. of apnea	Mean durationof hypopnea	No. ofdesaturations	% time Sat < 90%	% time Snoring
ESS	− *.208*	.104	.059	.047	.069	.049	− .038	− .010	− .019	.034	− .025	.040

The significance is 5%

Among the group of men, a significant correlation was found between ESS to BMI (*r* = .107, *p* = .010), AHI (*r* = .158, *p* = .000), number of apneas (*r* = .129, *p* = .002), number of hypopneas (*r* = .115, *p* = .006), number of blood oxygen desaturations (*r* = .145, *p* = .000), and percentage of the time blood oxygen saturation was under 90% (*r* = .130, *p* = .002). However, among the group of women, no significant correlation was found between any of the sleep test parameters or BMI and ESS. Nevertheless, among the women’s group, a negative correlation was found between age and EDS (*r* = − .208, *p* = .000), suggesting that young women are more tired than elderly women.

Table 4 shows differences between age groups, women under and above 50 years of age.

**Table 4:** Women, age groups. Correlations between ESS to sleep study variables

	BMI	No. of apneas	No. of hypopneas	AHI	Max. dur. of apnea	Max. dur. of hypopnea	Mean dur. of apnea	Mean duration of hypopnea	No. of desaturations	% time Sat < 90%	% time Snoring
Women < 50	.034	.219	− .029	.150	.098	− .144	.051	.023	.052	.078	− .039
Women > 50	.183	.019	.138	.080	.073	.122	− .007	− .023	.079	− .039	.110

Table 5 shows the results of an independent samples t-test that was conducted to examine gender differences in ESS scores. Levine’s test for equality of variances showed no violations (*p* = .235). Results indicated that men (*n* = 580, M = 8.43, SD = 4.786) do not have significantly different mean ESS than women (*n* = 278, M = 8.05, SD = 4.549). *t*(856) = 1.108, *p* = .268.

**Table 5:** *T*-test for difference in ESS score among males and females

Independent sample test										
		Levene’s test for equality of variances				*T*-test for equality of means				
		*F*	Sig.	*t*	df	Sig. (2-tailed)	Mean difference	Std. Error difference	95% confidence interval of	
									Lower	Upper
ESS	Equal variances assumed	1.412	.235	1.108	856	.268	.381	.344	− .294	1.413
	Equal variances not assumed			1.128	571.822	.260	.381	.338	− .282	1.414

## Discussion

Based on clinical observation and previous studies, men and women express different symptoms of OSAS and clinical symptoms predicting OSAS are more specific to men. Our goal for the present study was to characterize gender differences in the determinants of EDS among OSAS patients. In order to do so, we examined the correlation between sleep study variables and ESS, in the entire sample and separately in gender groups. This is the first study to examine the correlation between sleep study variables and ESS separately in men and women.

The gold standard test to examine EDS is MSLT. Since MSLT is a complicated, expensive test which includes a night and a day in a sleep lab, we used the ESS questionnaire. The ESS is the most widely used clinical tool for evaluating subjective sleepiness. It is considered a reliable and a validated method to estimate EDS. The use of ESS enabled us to have a large sample size in the study.

AHI is considered the main parameter to estimate OSAS severity. However, according to previous studies, AHI has shown conflicting results regarding the correlation between ESS and sleepiness. We therefore examined the correlation not only between AHI and ESS but also between several sleep study variables and ESS.

In order to enable comparison between the present study and previous studies, we first examined the correlation between sleep study parameters and ESS in the whole sample, without dividing the sample into gender groups. The results were similar to other studies that examined the correlation between sleep variables and ESS. Correlation was found between O_2_ desaturations, BMI, and AHI and ESS (Table 2). Previous studies that examined the correlation between sleep study parameters and ESS showed an association between hypoxemia, BMI, and AHI to EDS [7, 20–22]. Another study, by Gulbar et al., showed significant correlation between the duration of sleep apnea/hypopnea and EDS [21].

Having shown that the results of the entire sample are consistent with the results of previous studies, we examined the gender differences. We used the same methods on the sample divided to gender groups. According to our study’s results, the males’ group showed an association between the number of desaturations, percentage of time the blood saturation was under 90%, BMI and AHI and ESS (the same sleep parameters that correlated to ESS in the whole sample). Surprisingly, when we analyzed the females’ group, they did not show any significant correlation between sleep parameters or BMI to ESS. These results suggest that females’ EDS is not mainly influenced by sleep parameters. Previous studies showed that the prevalence of sleep-related breathing disorders and particularly OSA rises with menopause [22]. Therefore, we thought that the results might be explained by the difference between OSAS prevalence between young and older women. We did not assess menopausal status, which prevented us from directly examining any relationship of menopause with OSA and EDS. However, since women around 50 are likely to be in menopause, we divided the women’s group into two age groups, under the age of 50 and above the age of 50. In both age groups, we did not find any correlation between sleep variables or BMI and ESS. Another surprising finding supporting this result is that in the women’s group (undivided by age), we found a negative correlation between age and ESS, indicating that as opposed to expected, young women suffer from EDS more than elder women. Our study shows that despite increased prevalence of sleep-related breathing disorders among postmenopausal women, they do not suffer from EDS more than young women. Thus, EDS among postmenopausal women might not be a result of OSA. This finding strengthens our finding that breathing parameters are not correlated with EDS in women, as opposed to men.

An alternative hypothesis that could also explain the results is that men suffer from EDS more than women. In order to understand the results better, we examined whether there was a difference between sleepiness of men and women by comparing mean ESS scores between the gender groups. We found that mean ESS scores were not significantly different between men and women, indicating that there is no association between gender and degree of EDS, but the difference between genders is in the contributors to EDS.

The pathogenesis of sleepiness among patients with OSA appears to differ between men and women. While men’s sleepiness is influenced by BMI and breathing parameters, we could not find this connection in women. The reasons for the difference are unclear. A possible explanation is that women’s manifestations of OSA differ from the manifestations in men. Many previous studies have suggested that women with OSA more frequently report “atypical” symptoms of poor sleep quality, morning headaches, insomnia, and frequent awakening at night rather than EDS [11–16].

The importance of the results is in the understanding that women with OSA might not complain about EDS. According to our study’s results, EDS is not a predictive symptom of OSA among women. It is accepted that women with OSA are underdiagnosed. The underdiagnosis may be due to clinical manifestations that are less recognized by primary care physicians. The difference in the manifestations between the genders should be considered when screening for OSA.

Another issue stemming from our findings concerns OSA comorbidities. The difference between genders in the influence of OSA and sleep variables on EDS raises questions about other OSA results—is there a difference between genders regarding OSA comorbidities other than EDS? Further research is needed in this subject.

### Limitations

We used the ESS questionnaire, which is a validated tool to assess subjective EDS; however. the ESS is not the gold standard. Using MSLT for the evaluation of EDS might yield more accurate results.

We did not assess comorbidities of the patients that could contribute to sleepiness, such as hypothyroidism, CHF, COPD, and depression.

## Conclusion

This study was designed to research gender differences regarding potential contributing factors to EDS in OSAS patients. Our study showed that OSAS influences daytime sleepiness in men but not women. We showed that BMI and breathing parameters [AHI, O2] are associated with sleepiness in men, while the ESS of women with OSAS did not show correlation with breathing parameters or BMI.
